# High-sensitivity C-reactive protein is related to age and gender in an acute psychiatric inpatient population

**DOI:** 10.1016/j.heliyon.2022.e08992

**Published:** 2022-02-19

**Authors:** Yuki Sakai, Jeanette Brun Larsen, Solveig Klæbo Reitan

**Affiliations:** a UMR 1253, iBrain, Université de Tours, Inserm, Tours, France; b Norwegian University of Science and Technology, Faculty of Medicine and Health Science, Department of Mental Health, Trondheim, Norway; c St. Olav's University Hospital, Department of Mental Health, Trondheim, Norway

**Keywords:** CRP, Aging, Inflammation, Psychiatry, Immune markers, Emergency psychiatry, Mental disorders

## Abstract

Increased levels of high-sensitivity C-reactive protein (hsCRP) is associated with several psychiatric disorders. Demographic factors such as age and gender might affect this association, but the results are conflicting. The aim of this study was to explore a relationship between age, gender and hsCRP in an acute psychiatric inpatient population. We included 484 patients admitted to an acute psychiatric ward. Based on age distribution percentiles (25%, 50% and 75 %), we categorized patients into three age groups; ≦31 years old, 31–47 years old and ≧ 48 years old. Differences in serum levels of hsCRP between the age groups were assessed in the total sample, within males and females, and within diagnostic groups. There were significant differences in hsCRP across age groups. The effect was stronger in males than females. The significant differences between age groups were kept among patients with substance use disorders and bipolar disorders, but not among schizophrenia spectrum disorders, unipolar depression, neurotic disorders and personality disorders. Our findings suggest that the previously known association between age and hsCRP is present within an acute psychiatric population. However, this association was not found for all psychiatric diagnoses.

## 1 Introduction

C-reactive protein (CRP) is a widely used marker of inflammation of clinical importance especially in infection, but also in autoimmunity. Increase in low levels of CRP (measured as high-sensitivity CRP, hsCRP) is also gaining attention as a marker of cardiovascular risk. Importantly, hsCRP is used to reveal those under intermediate (CRP > 1 mg/L) and high (CRP > 3 mg/L) risk for cardiovascular events (Pearson et al., 2003; Ziv-Baran et al., 2017). The level of hsCRP also differs between genders, which might have consequences for the risk of cardiovascular diseases and cancer (Halcox et al., 2014; Li et al., 2017).

During the last decade, hsCRP has gained increased interest in psychiatry. Severe mental disorders such as schizophrenia, bipolar disorder and depression have been associated with higher levels of CRP (Dickerson et al., 2013; Fernandes et al., 2016; Haapakoski et al., 2015). The other way around, higher levels of hsCRP might also increase the risk for schizophrenia, bipolar disorder and depression (Wium-Andersen et al., 2016), which are comparable to the risk for somatic diseases (Wium-Andersen et al., 2014).

When comparing variation in hsCRP among different psychiatric disorders, it is important to be aware of confounding factors, of which age is one. Actually, *inflammaging* is a term used to describe the altered immune activity in old people (Deleidi et al., 2015; Rea et al., 2018). In summary, the pathological process of inflammaging is an interaction between several pillars (e.g. stress, epigenetics, macromolecular damage, metabolism), and inflammation is a fundamental driver of this interaction (Franceschi et al., 2018). Considering the immunological mechanisms, macrophages are central in creating a chronic activation of the innate immune system (Franceschi et al., 2000). More specifically, the process of aging also influences our macrophages with decreased ability to phagocytize, whereas production of pro-inflammatory cytokines increase (De Maeyer and Chambers, 2021). These cytokines can stimulate the liver into CRP production (Sproston and Ashworth, 2018).

Gender is also considered an important confounding factor when assessing changes in hsCRP (Liu et al., 2014; Woloshin and Schwartz, 2005). Several studies on psychiatric patients have found higher levels of hsCRP in females compared to males (Joseph et al., 2015; Wysokinski et al., 2015), but the results are conflicting (Osimo et al., 2018; Sanchez-Autet et al., 2018). The risk for psychiatric disorders known to be associated with higher hsCRP, might also differ between males and females (Liu et al., 2014; Liukkonen et al., 2006).

Findings are deviating when studying the effect of hsCRP on age in psychiatric patients (Haapakoski et al., 2015; Joseph et al., 2015). In addition, only one study has investigated the effect of age and gender on hsCRP on a group of patients admitted to an acute psychiatric department (Osimo et al., 2018). Osimo and colleagues found that hsCRP was associated with older age, but not gender, in an acute psychiatric population. However, more studies are needed in order to conclude on the subject. Inflammaging is related to several somatic age related diseases (Franceschi et al., 2018), but we still need more knowledge on how this interferes with psychiatric disorders. Thus, we analysed the level of hsCRP in different age groups and over different diagnostic groups and in different genders in a large acute psychiatric inpatient population.

## 2 Methods

### 2.1 Aims and hypotheses

The aim of this study was to explore a relationship between age and hsCRP in a general acute psychiatric inpatient population. A secondary aim was to explore gender differences in levels of hsCRP. Thirdly, we wanted to examine if there were any differences in these associations within different psychiatric disorders. We hypothesized that hsCRP would be related to both gender and age within an acute psychiatric population, and that this would be relevant for a variety of psychiatric disorders.

### 2.2 Design and setting of the study

This study is a cross sectional study. All patients admitted to the inpatient acute psychiatric ward, Department of Mental Health, St. Olav's University Hospital in Trondheim, Norway, from the end of 2004–2006 except vacations were eligible for inclusion (*n* = 834). Patients giving written informed consent were included.

### 2.3 Ethics

The study was approved by the Norwegian Regional Committee for Ethics (Identification number 199/04) and by Middle Norway and Norwegian Social Science Data Services (NSD) (No 11771). The study is registered at ClinicalTrials.gov (NCT00184418). Data collection and analysis were performed according to the Declaration of Helsinki.

### 2.4 Patients

In total 585 patients gave written informed consent to participate and were included (response rate: 70.14 %). Patients with immunologically related disorders were excluded from the present analyses (International Classification of Diseases version 10 (ICD-10): B17.1/18.2/18.9/20/24, C77.4, D50, E10/10.4/10.9/14, G35, K50/51.9, M35/79; *n* = 21) or presence of anti-thyroid peroxidase antibodies (*n* = 2). In addition, patients with missing data of CRP or hsCRP were excluded (*n* = 78). In total, we obtained data for 484 patients for further statistical analysis.

### 2.5 Diagnoses

Patients’ psychiatric diagnoses were set upon discharge according to ICD-10 Criteria for Research (WHO, 1993). Diagnoses were approved in a consensus meeting with at least two senior psychiatrists or clinical psychologists, of whom at least one had examined the patient personally. Diagnosis were categorized into 7 groups based on ICD-10 codes: substance use disorders (SUD: F10-19), schizophrenia spectrum disorder (SCZ: F20-29), bipolar disorders (F30–F31.5), unipolar depression (F32–F33), neurotic disorders (F40-49), personality disorders (F60-69) and all other diagnoses as 'others'. If multiple diagnoses were made, the primary diagnosis were used for further analysis.

### 2.6 Age groups

Based on age distribution percentiles (25%, 50% and 75 %), we categorized patients into three age groups; ≦31 years old, 31–47 years old and ≧ 48 years old.

### 2.7 Blood samples and laboratory assays

Blood samples were collected between 7:00 and 10:00 a.m. the first working day upon admission. The samples were handled according to standard procedures and analysed by Siemens Advia Chemistry XPT at the clinical laboratory at St Olav's University Hospital, Trondheim, Norway.

### 2.8 Statistics

The biological parameter hsCRP was included as a continuous variable. Diagnostic group, age groups and gender were categorical variables. Since the normality of hsCRP level assessed by the Kolmogorov–Smirnov test was skewed, non-parametric statistical analyses were chosen for further analysis.

We assessed differences in levels of hsCRP across age groups with a Kruskal-Wallis H-test, both in total and gender separated samples. We also examined if there was a difference in hsCRP level between male and female within each age group by a Mann-Whitney U Test. Additionally, we investigated the difference of hsCRP levels across the three age groups within each diagnostic category by a Kruskal-Wallis H-test.

If a statistically significance was indicated in any analysis, Dunn-Bonferroni correction was used for post-hoc test to avoid multiple test problems. In this statistical analysis, IBM-SPSS version 22 was used to assess all data. Significance level were set at < .05. All analyses were two-tailed.

## 3 Results

### 3.1 Demographic and clinical characteristics of the population

Demographic characteristics of patients including number of patients in each diagnostic group are given in Table 1.

**Table 1:** Characteristics of patients.

	Total (N = 484)	Male (N = 223)	Female (N = 261)
Age	39.0 (27.0–52.0)	38.0 (27.0–53.0)	40.0 (25.0–51.0)
Clinical Diagnoses			
SUD	74	45	29
SCZ	91	45	46
Bipolar Disorders	59	30	29
Unipolar Disorders	96	37	59
Neurotic Diorders	44	14	30
Personality Disorders	33	10	23
Other	87	42	45
hsCRP (mg/L)	1.71 (0,62–4.75)	1.67 (0.58–5.78)	1.74 (0.63-4-34)

Data are presented as median with interquartile range or n.

Abbreviations: hsCRP: high sensitivity C-reactive protein, SCZ: schizophrenia spectrum disorders, SUD: substance use disorders.

### 3.2 Level of hsCRP across age groups and genders

**Figure 1:**
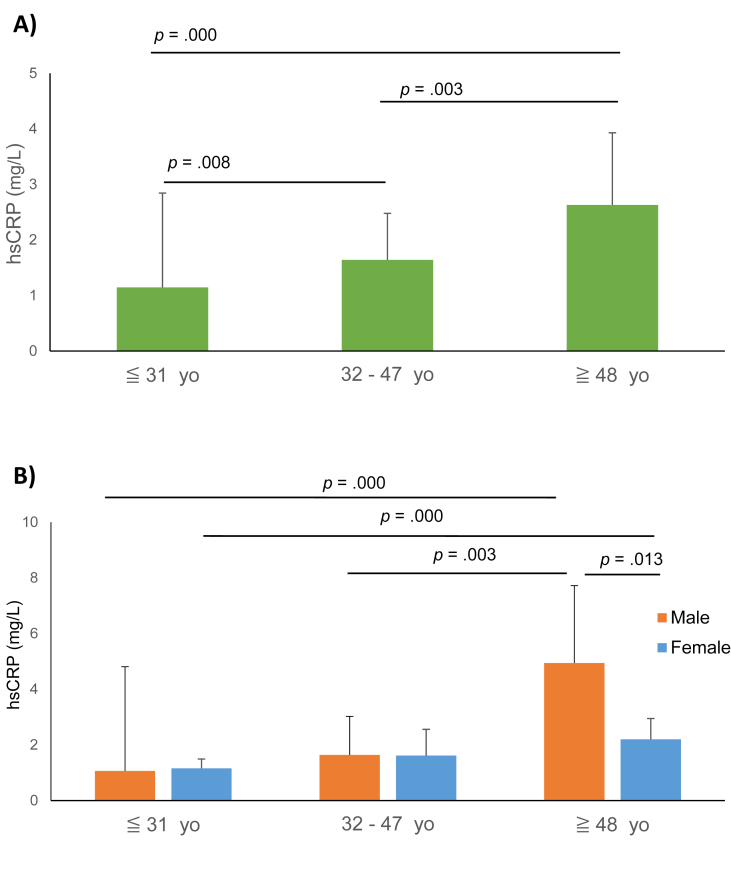
Differences in hsCRP levels among age groups. Part A shows results for the total sample, part B for gender separated sample. All p-values are obtained from Kruskal-Wallis H-test after post-hoc Dunn-Bonferroni correction. Abbreviations: hsCRP: high sensitivity C-reactive protein yo; years old.

There were significant differences in hsCRP across age groups (*x*^*2*^ (*df*) = 39.862 (2), *p* = .000) (Table 2). After post-hoc Dunn-Bonferroni correction, hsCRP levels were significantly higher in the oldest age group compared to the youngest group (*p* = .000) and the middle age group (*p* = .003). Similarly, the youngest age group had significantly lower hsCRP than both middle and oldest age group (*p* = .008) (Figure 1).

**Table 2:** Levels of hsCRP across different age groups (hsCRP (mg/L)).

	≦ 31 yo	32 - 47 yo	≧ 48 yo	*p-value*
Total (n)	164	161	159	
	1.15 (0.38–2.55)	1.64 (0.67–4.58)	2.63 (1.16-8-22)	***p* < 0.001**
Gender Separated				
Male (n)	74	81	68	
	1.07 (0.34–2.50)	1.64 (0.61–4.95)	4.94 (1.19–14.20)	***p* < 0.001**
Female (n)	90	80	91	
	1.16 (0.40-2-62)	1.62 (0.71–4.33)	2.20 (1.12-4-94)	***p* = 0.001**
Clinical Diagnosis				
SUD (n)	27	29	18	
	2.19 (0.62–4.34)	3.04 (0.79–5.92)	9.12 (2.01–12.13)	***p* = 0.047**
SCZ (n)	18	38	35	
	2.29 (0.45-5-51)	2.30 (0.73–5.52)	3.47 (1.41–13.45)	*p* = 0.169
Bipolar Disorders (n)	14	16	29	
	0.64 (0.31–1.92)	1.66 (0.61–3.49)	2.23 (1.27–5.23)	***p* = 0.013**
Unipolar Depression (n)	34	28	34	
	0.90 (0.32-2-32)	1.61 (0.71-3-52)	2.01 (0.84-3-55)	*p* = 0.065
Neurotic Diorders (n)	20	16	8	
	0.48 (0.31–1.95)	0.73 (0.56-1-63)	1.61 (0.36–7.14)	*p* = 0.180
Personality Disorders (n)	19	10	4	
	1.18 (0.40–2.48)	1,78 (0.82–8.47)	4,11 (0.47–11.92)	*p* = 0.433
Other (n)	32	24	31	
	1.11 (0.42–1.82)	1.11 (0.40–4.59)	3.66 (1.34–12.65)	***p* = 0.001**

Data are presented as n and median with interquartile range. P-values obtained by a Kruskal-Wallis H-test.

Abbreviations: hsCRP: high sensitivity C-reactive protein, SUD: substance use disorders, SCZ: schizophrenia spectrum disorders, yo = years old.

In the male sample, there were significant differences comparing all age groups (x^2^ (*df*) = 27.234 (2), *p* = .000) (Table 2). A pairwise post-hoc Dunn test with Bonferroni adjustment was significant for the oldest group compared to the middle age group (*p* = .003) and for the oldest group compared to the youngest (*p* = .000) (Figure 1). However, we found no significant differences between the middle and youngest age groups (*p* = .121). A significant difference between age groups was also found in female patients (*x*^*2*^ (*df*) = 14.774 (2), *p* = .001) (Table 2). However, here, hsCRP was significantly different only between the oldest and the youngest age groups (*p* = .000) (Figure 1).

Levels of hsCRP were not significantly different between males and females (*U* = 27708, *p* = .364) in the total sample (Table 3). Comparing the level of hsCRP between males and females in each of the three age groups showed a significant difference only in the oldest age group. Male patients aged 48 years and above had significantly higher hsCRP level than females in same age group (*U* = 2377, *p* = .013) (Table 3).

**Table 3:** Gender differences in hsCRP (mg/L).

	Male (N = 223)	Female (N = 261)	p-value
Total sample	1.67 (0.58–5.78)	1.74 (0.63-4-34)	p = 0.364
Age groups:			
≦ 31 yo	1.07 (0.34–2.50)	1.16 (0.40-2-62)	p = 0.851
32–47 yo	1.64 (0.61–4.95)	1.62 (0.71–4.33)	p = 0.976
≧ 48 yo	4.94 (1.19–14.20)	2.20 (1.12-4-94)	**p = 0.013**

Data are presented as median with interquartile range. P-values obtained by Mann-Whitney U Test.

Abbreviations: hsCRP: high sensitivity C-reactive protein, yo = years old.

### 3.3 Level of hsCRP across psychiatric diagnostic groups

Analyzing hsCRP levels across age groups within the different diagnostic groups, showed that the significant difference between age groups was kept among patients with SUD (*x*^*2*^ (*df*) = 6.095 (2), *p* = .047), bipolar disorders (*x*^*2*^ (*df*) = 8.687 (2), *p* = .013) and “other” (*x*^*2*^ (*df*) = 13.75 (2), *p* = .001) (Table 2). For patients with SCZ, unipolar depression, neurotic disorders and personality disorders, the effect of age did not reach significance, even though the sample size for the groups SCZ and unipolar depression was similar to the other groups (Table 2).

## 4 Discussion

We found an association between age and hsCRP in an acute psychiatric inpatient population. The percentile of patients aged 48 years and above had significantly higher levels of hsCRP compared to younger patients. In addition, there was a gradient towards lowest hsCRP in the lower percentile of patients aged 30 and below. The effect was stronger in males than females. When analyzing within different psychiatric diagnostic groups, we found the age effect in all groups apart from those with schizophrenia spectrum disorders, unipolar depression, neurotic disorder and personality disorder.

Aging is associated with increased baseline inflammation measured as hsCRP and pro-inflammatory cytokines (Daynes et al., 1993; Soysal et al., 2016). However, most previous studies have focused on the old age population (e.g. above 65 years old). For younger people reports are sparse. Thus, our data showing significant effects of age also among younger persons, though stronger in the older age groups, is interesting. The age effect is important to bear in mind analysing CRP in relation to diagnoses and symptom level.

Male gender was associated with increased hsCRP, especially in the oldest age group. The higher hsCRP in males compared to females is in line with a few other reports (Tayefi et al., 2017; Vetter et al., 2013). CRP is a risk factor for several disease, including cardiovascular disorders. Interestingly, psychiatric patients have a significantly increased risk of cardiovascular disorders and morbidity (Hayes et al., 2017; Ringen et al., 2014). It might be that inflammation and hsCRP is a common risk factor for cardiovascular and psychiatric diseases (Sforzini et al., 2019). Therefore, our finding of significantly increased hsCRP in older men is important to explore further.

Previous studies on certain psychiatric diagnostic groups have shown increased CRP compared to controls (Dickerson et al., 2013; Fernandes et al., 2016). However, few have studied the level of inflammation compared between different diagnostic groups. Comparing different diagnostic groups could aid in exploring which psychiatric disorders have increased inflammation as part of a general psychiatric stress, and which disorders involve more inflammation as part of its etiology or comorbidity. We now have been able to show that the age effect is seen in many patient groups – though there seems to be less effect of age among patients in the groups schizophrenia spectrum disorder, unipolar depression, neurotic disorders and personality disorders – a finding that need to be further explored.

In the groups' neurotic disorders and personality disorders this may have been due to loss of power with fewer participants. However, for the groups’ schizophrenia and unipolar depression, the number of participants was similar to or higher than the other groups. Inflammation is a rather consistent finding in unipolar depression (Tayefi et al., 2017) and in schizophrenia (Dickerson et al., 2013). Thus, it is possible that in these diagnostic groups, the inflammation related to the diagnoses (e.g. schizophrenia and depression) is masking the effect of age. It is interesting that inflammation related to diagnoses in these groups is so pronounced that it outlines the effect of age. Still, age should be taken into account in future studies in depression and schizophrenia.

There are several weaknesses to the current study. We did not correct for body mass index (BMI). As BMI is related to hsCRP (Groven et al., 2019; Santos et al., 2005), this must be considered a weakness. Furthermore, BMI alone may not be the main driver of increased CRP as it may be a marker for other factors driving the metabolic syndrome. Also, no data on medication was available. We did not have a healthy control group that could extend the interpretation of the study. However, considering that the primary aim of this study was to investigate the effect of age on hsCRP within a population of psychiatric patients; a healthy control group is not strictly necessary. Another limitation is that the study was not powered for testing the relation between hsCRP and age. Finally, the cross sectional design is a weakness and we can therefore not draw any causal conclusions between hsCRP and age.

It is a strength to the study that the sample size is rather high. In addition, it represents a broad specter of psychiatric patients included when they were severely and acutely ill. The population is heterogeneous and were included from a health system free of charge, excluding many socioeconomic confounders. All patients in the area needing acute psychiatric services were admitted to this ward.

## 5 Conclusions

The present study indicates an association between hsCRP and higher age in an acute psychiatric population. However, the association was only found within some diagnostic categories, indicating that there are other factors driving the inflammation where the association was lacking.

## Declarations

### Author contribution statement

Solveig Klæbo Reitan: Conceived and designed the experiments; Performed the experiments; Analyzed and interpreted the data; Contributed reagents, materials, analysis tools or data; Wrote the paper.

Yuki Sakai and Jeanette Brun: Analyzed and interpreted the data; Wrote the paper.

### Funding statement

This work was supported by 10.13039/100009123Norwegian University of Science and Technology, Department of Mental Health and St. Olav's University Hospital, Department of Mental Health.

### Data availability statement

Data will be made available on request.

### Declaration of interests statement

The authors declare no conflict of interest.

### Additional information

The clinical trial described in this paper was registered at ClinicalTrials.gov under the registration number NCT00184418.

## References

[bib1] Daynes R.A., Araneo B.A., Ershler W.B., Maloney C., Li G.Z., Ryu S.Y. (1993). Altered regulation of IL-6 production with normal aging. Possible linkage to the age-associated decline in dehydroepiandrosterone and its sulfated derivative. J. Immunol..

[bib2] De Maeyer R.P.H., Chambers E.S. (2021). The impact of ageing on monocytes and macrophages. Immunol. Lett..

[bib3] Deleidi M., Jaggle M., Rubino G. (2015). Immune aging, dysmetabolism, and inflammation in neurological diseases. Front. Neurosci..

[bib4] Dickerson F., Stallings C., Origoni A., Vaughan C., Khushalani S., Yang S., Yolken R. (2013). C-reactive protein is elevated in schizophrenia. Schizophr. Res..

[bib5] Fernandes B.S., Steiner J., Molendijk M.L., Dodd S., Nardin P., Goncalves C.A., Jacka F., Kohler C.A., Karmakar C., Carvalho A.F., Berk M. (2016). C-reactive protein concentrations across the mood spectrum in bipolar disorder: a systematic review and meta-analysis. Lancet Psychiatr..

[bib6] Franceschi C., Bonafè M., Valensin S., Olivieri F., De Luca M., Ottaviani E., De Benedictis G. (2000). Inflamm-aging. An evolutionary perspective on immunosenescence. Ann. N. Y. Acad. Sci..

[bib7] Franceschi C., Garagnani P., Parini P., Giuliani C., Santoro A. (2018). Inflammaging: a new immune-metabolic viewpoint for age-related diseases. Nat. Rev. Endocrinol..

[bib8] Groven N., Fors E.A., Reitan S.K. (2019). Patients with Fibromyalgia and Chronic Fatigue Syndrome show increased hsCRP compared to healthy controls. Brain Behav. Immun..

[bib9] Halcox J.P., Roy C., Tubach F., Banegas J.R., Dallongeville J., De Backer G., Guallar E., Sazova O., Medina J., Perk J., Steg P.G., Rodriguez-Artalejo F., Borghi C. (2014). C-reactive protein levels in patients at cardiovascular risk: EURIKA study. BMC Cardiovasc. Disord..

[bib10] Hayes J.F., Marston L., Walters K., King M.B., Osborn D.P.J. (2017). Mortality gap for people with bipolar disorder and schizophrenia: UK-based cohort study 2000-2014. Br. J. Psychiatry.

[bib11] Haapakoski R., Mathieu J., Ebmeier K.P., Alenius H., Kivimaki M. (2015). Cumulative meta-analysis of interleukins 6 and 1beta, tumour necrosis factor alpha and C-reactive protein in patients with major depressive disorder. Brain Behav. Immun..

[bib12] Joseph J., Depp C., Martin A.S., Daly R.E., Glorioso D.K., Palmer B.W., Jeste D.V. (2015). Associations of high sensitivity C-reactive protein levels in schizophrenia and comparison groups. Schizophr. Res..

[bib13] Li Y., Zhong X., Cheng G., Zhao C., Zhang L., Hong Y., Wan Q., He R., Wang Z. (2017). Hs-CRP and all-cause, cardiovascular, and cancer mortality risk: a meta-analysis. Atherosclerosis.

[bib14] Liu Y., Al-Sayegh H., Jabrah R., Wang W., Yan F., Zhang J. (2014). Association between C-reactive protein and depression: modulated by gender and mediated by body weight. Psychiatr. Res..

[bib15] Liukkonen T., Silvennoinen-Kassinen S., Jokelainen J., Rasanen P., Leinonen M., Meyer-Rochow V.B., Timonen M. (2006). The association between C-reactive protein levels and depression: results from the northern Finland 1966 birth cohort study. Biol. Psychiatr..

[bib16] Osimo E.F., Cardinal R.N., Jones P.B., Khandaker G.M. (2018). Prevalence and correlates of low-grade systemic inflammation in adult psychiatric inpatients: an electronic health record-based study. Psychoneuroendocrinology.

[bib17] Pearson T.A., Mensah G.A., Alexander R.W., Anderson J.L., Cannon R.O., Criqui M., Fadl Y.Y., Fortmann S.P., Hong Y., Myers G.L., Rifai N., Smith S.C., Taubert K., Tracy R.P., Vinicor F. (2003). Markers of inflammation and cardiovascular disease: application to clinical and public health practice: a statement for healthcare professionals from the Centers for Disease Control and Prevention and the American Heart Association. Circulation.

[bib18] Rea I.M., Gibson D.S., McGilligan V., McNerlan S.E., Alexander H.D., Ross O.A. (2018). Age and age-related diseases: role of inflammation triggers and cytokines. Front. Immunol..

[bib19] Ringen P.A., Engh J.A., Birkenaes A.B., Dieset I., Andreassen O.A. (2014). Increased mortality in schizophrenia due to cardiovascular disease - a non-systematic review of epidemiology, possible causes, and interventions. Front. Psychiatr..

[bib20] Sanchez-Autet M., Arranz B., Safont G., Sierra P., Garcia-Blanco A., de la Fuente L., Garriga M., Garcia-Portilla M.P. (2018). Gender differences in C-reactive protein and homocysteine modulation of cognitive performance and real-world functioning in bipolar disorder. J. Affect. Disord..

[bib21] Santos A.C., Lopes C., Guimaraes J.T., Barros H. (2005). Central obesity as a major determinant of increased high-sensitivity C-reactive protein in metabolic syndrome. Int. J. Obes..

[bib22] Sforzini L., Pariante C.M., Palacios J.E., Tylee A., Carvalho L.A., Viganò C.A., Nikkheslat N. (2019). Inflammation associated with coronary heart disease predicts onset of depression in a three-year prospective follow-up: a preliminary study. Brain Behav. Immun..

[bib23] Soysal P., Stubbs B., Lucato P., Luchini C., Solmi M., Peluso R., Sergi G., Isik A.T., Manzato E., Maggi S., Maggio M., Prina A.M., Cosco T.D., Wu Y.T., Veronese N. (2016). Inflammation and frailty in the elderly: a systematic review and meta-analysis. Ageing Res. Rev..

[bib24] Sproston N.R., Ashworth J.J. (2018). Role of C-reactive protein at sites of inflammation and infection. Front. Immunol..

[bib25] Tayefi M., Shafiee M., Kazemi-Bajestani S.M.R., Esmaeili H., Darroudi S., Khakpouri S., Mohammadi M., Ghaneifar Z., Azarpajouh M.R., Moohebati M., Heidari-Bakavoli A., Parizadeh M.R., Nematy M., Safarian M., Ebrahimi M., Ferns G.A., Mokhber N., Ghayour-Mobarhan M. (2017). Depression and anxiety both associate with serum level of hs-CRP: a gender-stratified analysis in a population-based study. Psychoneuroendocrinology.

[bib26] Vetter M.L., Wadden T.A., Vinnard C., Moore R.H., Khan Z., Volger S., Sarwer D.B., Faulconbridge L.F. (2013). Gender differences in the relationship between symptoms of depression and high-sensitivity CRP. Int. J. Obes..

[bib27] WHO (1993).

[bib28] Wium-Andersen M.K., Orsted D.D., Nordestgaard B.G. (2014). Elevated C-reactive protein, depression, somatic diseases, and all-cause mortality: a mendelian randomization study. Biol. Psychiatr..

[bib29] Wium-Andersen M.K., Orsted D.D., Nordestgaard B.G. (2016). Elevated C-reactive protein and late-onset bipolar disorder in 78 809 individuals from the general population. Br. J. Psychiatry.

[bib30] Woloshin S., Schwartz L.M. (2005). Distribution of C-reactive protein values in the United States. N. Engl. J. Med..

[bib31] Wysokinski A., Margulska A., Strzelecki D., Kloszewska I. (2015). Levels of C-reactive protein (CRP) in patients with schizophrenia, unipolar depression and bipolar disorder. Nord. J. Psychiatr..

[bib32] Ziv-Baran T., Shenhar-Tsarfaty S., Etz-Hadar I., Goldiner I., Gottreich A., Alcalay Y., Zeltser D., Shapira I., Angel Y., Friedensohn L., Ehrenwald M., Berliner S., Rogowski O. (2017). The ability of the wide range CRP assay to classify individuals with low grade inflammation into cardiovascular risk groups. Clin. Chim. Acta.

